# Diabetic retinopathy: everybody's business

**Published:** 2011-09

**Authors:** Iris Winter, David Yorston

**Affiliations:** Consultant, Universitätsklinik und Poliklinik für Augenheilkunde/Department of Ophthalmology at University Hospital, Halle (Saale), Ernst-Grube Str. 40, 06120 Halle (Saale). Email: winteriris02@yahoo.com; Consultant Ophthalmologist, Tennent Institute of Ophthalmology, Gartnavel Hospital, 1053 Great Western Road, Glasgow G12 0YN, UK

**Figure:**
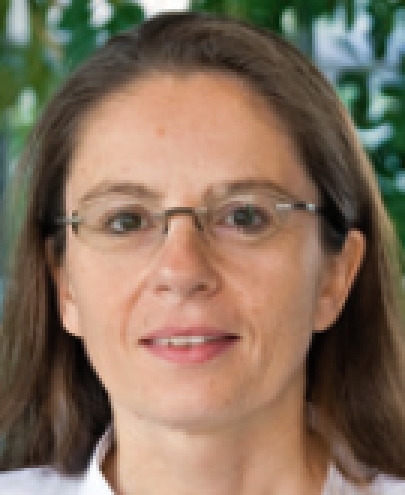


**Figure:**
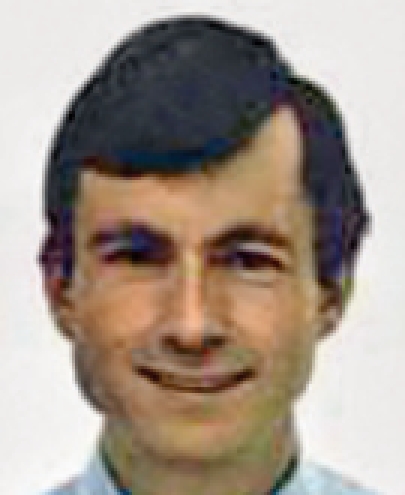


Diabetes is on the increase worldwide, due mainly to the rise in the number of people with type 2 diabetes. Type 2 diabetes is becoming more common because:

- People are living longer, and diabetes is more prevalent in older people.
- As people increasingly migrate to urban areas, exercise less, eat more, and eat less healthy food, more people are becoming obese - a primary cause of type 2 diabetes.

Diabetes increases the risk of a range of eye diseases, including cataract, but the main cause of blindness associated with diabetes is diabetic retinopathy (DR). DR usually develops between ten and twenty years after the onset of diabetes, and develops faster when diabetes is undiagnosed and untreated.

People with DR whose sight is at risk can be treated, most commonly with laser, to prevent visual impairment and blindness. Sadly, there is no treatment that can restore vision that has already been lost.

In 2030, the number of people with diabetes is expected to increase to 440 million, 54% more than in 2010. This means that, for every two people with diabetes today, there would be three in 2030. But there will be a far greater increase in some of the world's poorest regions (Table 1, overleaf). In sub-Saharan Africa, for example, the expected increase is 98%, which means the number of people with diabetes there would double.

**Figure:**
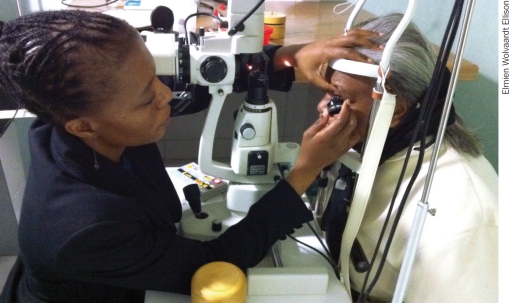
Laser is the most effective treatment for proliferative diabetic retinopathy. SOUTH AFRICA

As the prevalence of diabetes increases, so will the risk of DR. In 2002, the global average risk of blindness from DR amongst people with diabetes was calculated as 0.75% - meaning that, out of every 133 people with diabetes, one person will go blind. If we simply apply this statistic to the expected number of people predicted to have diabetes in 2030 (440 million), the number of people likely to go blind from DR would be 3.3 million.

In the poorest regions, however, the average risk of blindness from DR tends to be higher than 1 in 133. An important reason for this is that the infrastructure and resources required to effectively address DR are either inadequate or absent. For example, in one recent survey in Central America, 1.2% of people with diabetes were blind from DR (around 1 in 84).

In this respect, the current projections of the future global increase in blindness from DR appear optimistic, and the number people likely to become blind as a result of DR in 2030 could be much higher than 3.3 million.

Fortunately, because of the ten- to twenty-year delay in the onset of DR, we still have a small window of opportunity to put systems, equipment, and people in place now to cope with, and wherever possible prevent, the epidemic of DR that is likely to affect the poorest countries a decade or so from now.

## What can we do?

Many readers of this journal are ophthalmic nurses or medical assistants, and some may feel that DR is a complicated problem which should be left to programme managers or ophthalmologists to address. However, there is a great deal that all eye care workers and their health care colleagues can do to address DR, by supporting each of the following:

- prevention of diabetes
- early diagnosis of diabetes
- good control of blood sugar and blood pressure amongst people with diabetes
- early diagnosis of DR
- referral for treatment.

**Prevention of diabetes**. At the primary care level, we can emphasise the importance of a healthy diet and exercise, since much of the increase in diabetes is a result of increasing obesity. Eye workers should join with other health workers to emphasise that diabetes - and diabetic retinopathy - can be avoided by making healthier lifestyle choices.

**‘Diabetes — and diabetic retinopathy — can be avoided by making healthier lifestyle choices’**

**Early diagnosis of diabetes**. The people who are blind from DR today are the same people whose diabetes was undiagnosed or poorly controlled ten years ago. We know that early diagnosis of diabetes followed by good control of blood sugar and blood pressure will reduce the incidence of DR (page 4). However, many people do not know they have diabetes: the recent Nigeria national blindness and visual impairment survey found that nearly half of all participants with high blood sugar did not know they had diabetes.

**Table 1:** Expected increase in number of people with diabetes: 2010-2030

	2010		2030			
	Number of people with diabetes	Prevalence of diabetes	Number of people with diabetes	Prevalence of diabetes	Increase in number of people with diabetes
Region	Millions	%	Millions	%	%
Europe	55.2	6.9	66.2	8.1	20.0
North America and Caribbean	37.4	10.2	53.2	12.1	42.4
Middle East and North Africa	26.6	9.3	51.7	10.8	93.9
South and Central America	18	6.6	29.6	7.8	65.1
Western Pacific (incl. China)	76.7	4.7	112.8	5.7	47.0
Southeast Asia (incl. India)	58.7	7.6	101	9.1	72.1
Sub-Saharan Africa	12.1	3.8	23.9	4.7	98.1
**Total (average)**	**284.6**	**(6.4)**	**438.4**	**(7.7)**	**54.0**

Courtesy of Zoe Okrim. Data taken from IDF Diabetes Atlas 4th Ed, © International Diabetes Federation 2009

**Figure:**
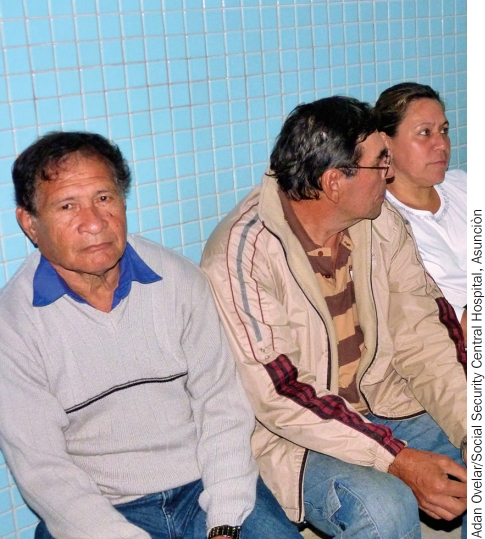
All diabetes patients must have a retinal examination every year. PARAGUAY

Where there are systems in place to manage diabetes and its complications, we must therefore do more to look for people with diabetes and improve their care (page 16). One example would be to do routine urine or blood glucose testing on all patients who present with cataract.

**Good control of blood sugar and blood pressure**. Even though improving a patient's control of blood sugar and blood pressure may be outside our area of expertise, we can advise our patients about the importance of good control and support them by ensuring that we are working in partnership with physicians and diabetes specialists. We should use every opportunity to reinforce the message that good control in the present will pay off in the future.

**Early diagnosis of DR**. Where there is treatment available for DR, all health care workers must reinforce the following messages when working with diabetes patients:

- Diabetes can cause blindness
- The early stages are only detectable by examination of the retina
- Everyone with diabetes should have an annual retinal examination to allow early diagnosis and treatment.

If all of us used every contact with every diabetes patient to reinforce these messages, then we can be confident that there would be a reduction in the risk of blindness due to DR.

**Referral for treatment**. For ophthalmologists and health planners, there is an urgent need to ensure that networks and resources for the referral and treatment of DR are in place. It is impossible and unnecessary to provide a laser in every eye clinic. However, every eye clinic needs to know where to send their patients who need laser treatment. Ophthalmology residency programmes should ensure that their curricula emphasise the conditions that will be the major causes of blindness in the future, such as DR. Although not all ophthalmologists will treat DR, they must all know how to recognise it and when to refer patients for treatment.

## Thinking beyond the clinic

One of the challenges in managing DR is that it requires partnerships with many different health care workers — both to find people with diabetes and to provide the eye care they need. We have to forge alliances with physicians, podiatrists, dieticians, pharmacists and all the other health workers and policy makers involved in the care of patients with diabetes. We will depend on them to encourage their patients to have annual eye examinations and we must return the favour by encouraging the patients we see to maintain good control of their diabetes and blood pressure. Every eye health worker can play a part in reaching out to other health workers and building the networks that will be critically important for preventing blindness from DR.

In this issue we have tried to make the complexities of DR relatively simple, so that all eye health workers will have a clearer idea of what DR is and what it looks like. We hope that the relatively simple DR grading system on page 12 will help you to decide who has sight-threatening DR and who has not. We have also tried to give clear guidelines for management, based on the best available evidence (page 5).

There is no single solution to DR that can be applied to every community worldwide. As with VISION 2020, the best results will be achieved by developing programmes at district level that take into account local conditions and resources. We have included some guidelines and a case study of a diabetes programme (page 17), not as a blueprint to be followed in every detail, but to help you to think about how you might achieve the same things in your own district, clinic, or village.

If there is one single message in this issue of the journal, it is this: diabetes and DR are everybody's business. We must not leave it just to the specialists, whether they are specialists in diabetes or in retinal disease. In future, every single health worker will have to contribute to preventing, detecting, and managing diabetes and diabetic retinopathy.

**Understanding diabetes and diabetic retinopathy**

Blood sugar levels are controlled by insulin, a hormone secreted by cells in the pancreas. In diabetes, this control mechanism breaks down, which leads to high levels of glucose in the blood. Type 1 diabetes is uncommon. It is caused by destruction of the insulin-secreting cells in the pancreas, and at present there is no means of predicting or preventing it. It occurs in young people and begins suddenly. This type of diabetes always requires treatment with injections of insulin.

Type 2 diabetes is much more common. It begins very gradually and may be completely without symptoms. Until recently, it was thought to affect only people over 40 years old, but it is now found in much younger patients, particularly in association with obesity. In most people, type 2 diabetes is related to obesity and may be prevented and often controlled by weight loss and exercise. Not all people with type 2 diabetes will require insulin. Some may be treated with tablets and some may only require weight loss to restore control of blood sugar.

Both type 1 and type 2 diabetes are serious conditions. In both forms, the elevated blood sugar causes complications, most of which are due to damage to small blood vessels. This causes diabetic retinopathy, kidney disease, and foot ulceration — which may lead to amputation. In addition, the high blood sugar increases the risk of blockage of larger blood vessels, leading to strokes and heart attacks.

In **diabetic retinopathy (DR)**, damaged small blood vessels leak in the retina at the back of the eye. Later, the blood vessels become blocked, which leads to the formation of abnormal new blood vessels. These new vessels are fragile and can bleed into the vitreous gel; they can also pull on the retina, causing retinal detachment. If blood vessels become damaged in the central part of the retina, this causes diabetic maculopathy, which is characterised by swelling of the retina (macular oedema).

All of these changes can damage vision permanently, and eventually lead to blindness unless the patient receives treatment (mainly laser treatment). Even then, treatment will only stop or slow down the disease — existing damage to the eye or to the patient's vision cannot be undone.

Everyone with diabetes will develop some degree of DR eventually, most commonly after ten or more years of living with diabetes. High blood sugar and high blood pressure increases the risk of developing DR.

